# What Western Philosophers of Technology Might Learn from Li Bocong’s Philosophy of Engineering

**DOI:** 10.1007/s11948-024-00490-4

**Published:** 2024-07-23

**Authors:** Nan Wang, Carl Mitcham

**Affiliations:** 1 University of Chinese Academy of Science, No. 1 Yanqihu East Rd, Huairou District, Beijing, 101408 People’s Republic of China; 2 Colorado School of Mines, 1500 Illinois St., Golden, CO 80401 USA

**Keywords:** Chinese philosophy, Engineering ethics, Philosophy of engineering, Philosophy of technology, Li Bocong, Sociology of engineering

## Abstract

This essay aims to rectify a failure on the part of Western philosophers of technology to attend to the creative philosophical work of Li Bocong at the University of Chinese Academy of Sciences in Beijing. After a brief account of Li Bocong’s personal contacts with the West and some remarks on his relationship to Marxism, we take up three aspects of his philosophy that can contribute to enlarging Western philosophical thinking about engineering and technology: (1) Li’s analysis of engineering as more than design, (2) his argument for the relevance of the sociology of engineering, and (3) his conceptualization of engineering ethics as more than professional ethics.

## Introduction

Philosophy of technology and philosophy of engineering have become recognized fields in philosophy, but literature in these fields is weak on contributions from Chinese scholars. *The Oxford Handbook of Philosophy of Technology* (Vallor, 2022) includes a chapter on the relevance of Confucianism for the ethics of technology (Wong, 2022) but does not reference more general Chinese work in the philosophy of technology and engineering. *The Routledge Handbook of the Philosophy of Engineering* (Michelfelder & Doorn, 2021) includes an article on “Eastern Philosophical Approaches and Engineering,” which has a slight 3.5 pages on Chinese approaches; the Chinese section describes Li Bocong “as the founding father of the philosophy of engineering China” and someone who “made the first comprehensive effort to study philosophy of engineering” (Miller et al., 2021, p. 51) but does not go into the content of Li’s thought. Our essay aims to rectify such omissions with an introduction to some distinctive features of Li’s philosophical engagement with engineering and technology that Western philosophers of engineering and technology would do well to consider. There are, of course, more than the selective few we will highlight. But on the basis of our complementary knowledge and experience—of collaborating Chinese and American philosophers—we judge those on which we will focus to be especially pertinent.

Collaboration here has been particularly crucial. Since the American co-author does not know Chinese, he has necessarily been mentored by the Chinese co-author. When the Chinese co-author, as a former doctoral student of Li Bocong, spent two years as a postdoctoral researcher in the United States with the American co-author, they were able to develop a shared sense of differences between Chinese and American approaches to philosophical questions about technology. Our article also seeks to exemplify the kind of cross-cultural thinking that Li has practiced. No other Chinese philosopher of technology has done so much work both learning from and criticizing Western philosophy of technology. Indeed, Li probably knows more about Western philosophy of technology than any Western philosopher of technology knows about China.

After a brief account of Li Bocong’s personal contacts with the West and some remarks on his relationship to Marxism, we will take up three aspects of his philosophy that we think can make important contributions to Western philosophical thinking about engineering and technology: (1) his analysis of engineering as more than design, (2) his argument for the relevance of the sociology of engineering, and (3) his conceptualization of engineering ethics as more than professional ethics.

## Encounters and Comparisons with the West

Li Bocong began his research career in the 1970s focusing on traditional Chinese medicine, criticizing crude interpretations of five element theory and drawing on cybernetics, information theory, and systems theory (see Li, 1990). After the Reform and Opening he pursued advanced studies at the Graduate University of Chinese Academy of Sciences focusing on the history and philosophy of science, eventually shifting to an emphasis on philosophy, and then from philosophy of science to philosophy of technology, emphasizing the central importance of engineering. Reflecting his commitment to the Marxist philosophical tradition, however, Li’s philosophy of engineering and technology exhibits a strong affinity with what in the West goes under the name of “Science, Technology, and Society” (STS) studies. The Chinese modernization program since 1949, especially after the clarification of the Reform and Opening in 1978, challenged philosophy to recognize not just the problems of technology (as in early STS studies) but also its great benefits.

Li Bocong first directly met with Western philosophers of technology in 1992, when Carl Mitcham (from Pennsylvania State University) and Stephen Cutcliffe (from Lehigh University) were invited by the Chinese Academy of Social Sciences to give a series of talks on the development and scope of STS studies in the United States. The talks took place in Beijing at the Graduate University of Chinese Academy of Sciences where Li was a member of the humanities and social science faculty.

Subsequent to that initial meeting, Li Bocong spent the academic year 1994–1995 as a visiting scholar at Lehigh University and visited Pennsylvania State University. While in the United States, he participated in a meeting of the eighth biannual conference of the Society for Philosophy and Technology (SPT), held in 1995 at Hofstra University; there he shared his thesis regarding the philosophical importance of engineering, which had been published in China two years before (Li, 1993). From that point forward Li was a regular participant in SPT conferences and in associated conferences that became known as the Forum on Philosophy, Engineering, and Technology (fPET). Over the next 25 years Li’s articles began to appear in English, but because of their scattered and occasional character, often compounded by in inadequate translation, the significance of his thought was slow to be broadly appreciated in the West.

At the 2011 SPT meeting at the University of North Texas, Mitcham, as a member of the editorial board of the “Philosophy of Engineering and Technology” publication series, proposed that the series undertake to publish a book by Li Bocong. The initial idea was to translate his 2002 book *Gongcheng zhexue yinlun*工程哲学引论 (Introduction to philosophy of engineering). But because of its length (over 450 pages), subsequent developments in Li’s thinking, and a desire to relate his ideas more to Western philosophy, it was decided instead to take selections from the book while including modest supplementary material. In 2021, after a decade of work selecting materials and translation work led by Wang Nan, Li’s *An Introduction to Philosophy of Engineering* was finally published. For the first time it became possible for non-Chinese readers to engaged significantly with his work.

One of the defining arguments of that book is the recognition of engineering as central to any philosophy of technology. Li Bocong was among the first philosophers to make this argument. At the same time, as he was also the first to observe, there has been a parallel, independent emergence of engineering as a theme for scholarly reflection as well as practice—often in the form of collaborations among engineers, historians, sociologists, and philosophers—with Chinese scholars in a leadership role (Wang & Li, 2023). Indeed, Li’s analysis has emerged from extensive dialogue with the Chinese engineering community, including members of the Chinese Academy of Engineering (CAE). No other national academy of engineering has sponsored philosophical reflection to the same extent as the Chinese CAE. In no other country have professional engineering associations sought similar formal engagements with philosophers. The only possible exception is the collaboration between engineers and philosophers initiated after World War II by the Verein Deutscher Ingenieure (Association of German Engineers). The model and fruitfulness of such engagements is surely one element that Western philosophers of technology might take away from Li’s work.

## Engineering as More than Design

English-speaking engineers have classically defined engineering as “the art of directing the great sources of power in nature for the use and convenience of man” (to quote the 1828 Charter of the British Institution of Civil Engineers). Knowledge about the “sources of power in nature” was thought to be provided by science; hence the need to include physics, chemistry, and other sciences in an engineering education. The meaning of “use and convenience” was assumed to be obvious or able to be determined by the market; because “use and convenience” is largely determined by factors external to engineering practice, not much about this needed to be included in engineering education. The key component for English-speaking engineering has been the “art of directing,” which has generally been conceived, in the West, as a process of designing, or more specifically “engineering design” (Mitcham, 2020).

According to Li Bocong, engineering involves much more than technical design processes. It includes the whole nexus of practical activities and processes by which humans transform the natural world into one fit for human habitation. It is a particular kind of practice, especially productive and creative, which can be distinguished from cognitive activity.

When looking back at the history of philosophy in the West, Li Bocong notes (along with many others) how Descartes shifted the focus of philosophy from ontology to epistemology, from “what the world is” to “how to know the world.” But such philosophers, as Marx declared in the “Theses on Feuerbach,” have only interpreted the world in various ways, whereas the point is to change it. Now is the time to shift from epistemology to a theory of construction, i.e., to a focus not so much on how to know as how to change, construct, and create the world. This shift means breaking down epistemological barriers and exploring new areas for philosophy; what is needed is not new paradigms in epistemology but the creation of philosophy of engineering. The philosophy of engineering should replace the Cartesian maxim “I think, therefore I am” with a new one: “I create, therefore I am” (Li Bocong, 1993, 2021, pp. 204–207).

Although design remains a crucial part of engineering, Li Bocong’s philosophy of engineering does not limit itself to design. Instead, by distinguishing three stages in engineering activity—stages that include design, construction, operation, maintenance, and utilization—an analysis of engineering activity can build up a conceptual framework for the philosophy of engineering from the perspective of creative construction. For Li, engineering is only in part the design and creation of things; more comprehensively, it is the construction of large projects or what others have called socio-technical systems (Vermaas et al., 2011). (“Creative construction” here alludes to and contrasts with Joseph Schumpeter’s description of capitalism as “creative destruction”).

- The first stage of engineering activity (Li, 2021, chapter 2) is planning in accordance with certain purposes or aims, which is distinct from the formula of “practice-cognition-practice” in Marxist philosophy, with which most Chinese are familiar. Planning, purpose, or aim naturally become core categories for philosophy of engineering. When working out the plan for an engineering activity, engineers will be constrained by various conditions. Related concepts such as purposes, initial conditions, boundary conditions, anticipated consequences, opportunities, management, decision making, ideals, ideas, and rationality all occur in Li’s analysis as fundamental to the philosophy of engineering.
- Implementation is the essential and central stage of engineering activity (Li, 2021, chapter 3). Planning is a significant activity, but without operational implementation, however wonderful and thoughtful plans are just a mirage in the desert. The implementation of engineering activities is carried out through a set of operations done by certain practitioners in the light of the procedures defined by planning. Operation is definitely a key category in the philosophy of engineering. For Li Bocong, one of the distinguishing characteristics of modern engineering operations, in comparison with ancient ones, is that modern activities are mainly not completed by individuals but by collectives. Conceptions of organization, institution, management, leadership, duty, obligation, and more thus become important. In addition, some terms that Li uses to analyze implementation are manufacture, natural resources, raw stock, machines, products, wastes, labor, efficiency, control, feedback, and so on.
- A third stage implicating engineering activity is consumption and usage (Li, 2021, chapter 4). The dialectical relation between production and consumption, as analyzed by Marx in the preface to *The Critique of Political Economy*, points out that the stage of consuming, under which product potential is realized, is significant and necessary. Notions like consumption, exchange, trade, and life, in connection with consumption, are thus important in the philosophy of engineering. The ultimate goal or highest level of engineering activity is the realization of human freedom. But whether the original goal can ultimately be achieved goes beyond human predictive capabilities, especially in an increasingly complicated and rapidly changing modern society. These questions introduce further important terms into the philosophy of engineering: freedom, result, alienation, way of life, and more.

Engineering creative construction takes place, Li Bocong further notes, at different levels. In economics, for instance, there is a common distinction between micro- and macro-levels, and in the related field of business ethics there has been discussion of the importance of a meso-level. In like manner, Li argues that engineering creative construction takes place at the micro-level (individuals and enterprises), the meso-level (regions or industries), and the macro-level (nations and globally).

At all three levels, Li Bocong argues, engineering construction is a subclass of social construction. Referencing sociologists Berger and Luckmann (1966) and philosopher Searle (1995), Li suggests that engineering be analyzed as “the social construction of engineering reality.” For Li the term “social” can be further interpreted in narrow or broad senses. In a narrow sense the social implicates the economic, technological, psychological, or other dimensions of engineering activity. In a broad sense it integrates all these dimensions and is often replaced with the term “societal.” From another perspective the social construction of engineering takes place at the micro, meso, and macro levels.

For Li Bocong the levels analysis also suggests the need for some qualification of the “I” in the “I create, therefore I am” maxim. Searle (1995) and Tuomela (2003), for instance, have reflected on the status of personal pronouns. Searle distinguishes between “I intentions” and “we intentions”; Tuomela comments on the relationship between “I-mode” and “we-mode.” For both, all genuine social behavior contains collective intentionality on the part of the participants. According to Li, in the philosophy of engineering, as well, the creative constructor “I” can function as a member of an engineering community and as a “we” representative of an engineering community such as an enterprise or a department of an engineering project.

## Sociology of the Engineering as Community

For Li Bocong, the fact that modern engineering activity is the activity not simply of individuals but of groups suggests the need for a sociology of engineering as a complement to the philosophy of engineering. The situation is similar to the way sociology has also become increasingly salient in the philosophy of science.

Li Bocong pioneered Chinese development not only of the philosophy of engineering but also of the history and sociology of engineering, both of which he argues are crucial complements to philosophy. Adapting Imre Lakatos’ well-known thesis about the philosophy of science—that philosophy of science without history of science is empty and history of science without philosophy of science is blind—Li proposes that the philosophy of engineering without the history of engineering is empty and the history of engineering without the philosophy of engineering is blind (Li, 2018). Additionally, just as the history of science is necessarily a history of the scientific community, so the history of engineering will also be a history of the engineering community.

As is well known, Thomas Kuhn’s *The Structure of Scientific Revolutions* introduced the notion of “paradigm” into the philosophy of science, which in later work was grounded in that of “scientific community.” As Kuhn wrote in the second edition of his influential analysis, “if this book were being rewritten, it would … open with a discussion of the community structure of science” (Kuhn, 1970, p. 176). In fact, already in the mid-1930s the founder of the sociology of science, Robert K. Merton, had begun to focus on the social structure of the scientific community. Since then, the notion of a “scientific community” has been increasingly recognized as significant and has been used as well in studying the history and philosophy of science. The sociology of engineering, however, remains to be fully developed, as has the concept of an engineering community.

One way to characterize the engineering community is by comparison with the scientific community. According to Kuhn (1970), the basic goal of the scientific community is a search for truth. Furthermore, scientists form a homogenous group. Li Bocong argues that the engineering community has different goals and is heterogenous. Instead of truth it pursues value, from the value objective of productive forces to value in a broad sense. Additionally, various stakeholders besides engineers will contribute to engineering activity at different stages. In Li Bocong’s words, “The engineering community is composed of engineers, workers, investors, managers, and other stakeholders …. As a result, it is a heterogeneous community” (Li, 2010, p. 27). As heterogeneous, the engineering community involves strong differentiation. Not all members have undergone similar educations and professional initiations.

If the engineering community were a military unit, workers, managers, engineers and investors would be soldiers, commanders, staff officers, and directors of support services, respectively. There is another more vivid metaphor for the comparison of individual functions. If engineering is likened to a tank or bulldozer, then workers, managers, engineers and investors may also be compared to the canon or blade, steering wheel, engine, and fuel tank or fuels respectively. Every part is indispensable to the functional operation of the complete machine. (Li, 2010, p. 27)

The image that Li Bocong uses here (2010) is both distinctive and revealing. Li conceives the engineering community as one that transforms the physical world. This transformation can be destructive (like a tank) or constructive (like a bulldozer). But military destruction is always in the name of or prefatory to some larger construction. As has been emphasized, for Li engineering is not simply invention or design but involves creative action with a broader scope and implementation in the world. This difference further emphasizes the extent to which the engineering community is characterized by a complexity of internal relations among its members and by external relations with the environment.

Given its homogenous character, the scientific community as a whole (as well as particular communities such as physicists, chemists, and biologists) is readily accepted by non-scientists as the arbiter of reliable knowledge. Insofar as this is the case, Li Bocong argues that the relation between scientific communities and their societal contexts can be less problematic than that between engineering and its societal contexts (2010). The heterogeneity within the engineering community means that in comparison with the scientific community, engineering communities can manifest greater difficulties in resolving disputes, and there can be greater tensions between engineers and their social or societal contexts. In engineering communities there exist more kinds and degrees of division and cooperation, coordination and conflict, leadership and obedience, trust and distrust. “Similarly, there also exist coordination, cooperation, clash and conflict between the community and its environment” (Li, 2010, p. 2).

Li Bocong further suggests distinguishing between two types of engineering community: engineering professional communities and activity communities. Only a few engineers are formal members of professional engineering societies. Labor unions, professional societies, entrepreneurial associations, and the like all constitute communities and tend to actively defend their particular community interests as stakeholders. Groups of workers, engineers, investors, and managers that cooperate and engage in engineering projects constitute activity communities. Importantly, there can be tensions as well between these two types of engineering communities. For instance, technical engineering communities, worker communities, and investor communities often disagree about how much design effort and money to spend on project safety.

## Engineering Ethics as More Than Professional Ethics

Given his expansive understanding of engineering as creative construction activities in heterogenous communities of stakeholders, for Li Bocong, engineering ethics is obviously more than the ethics of a technical profession as defined in Western engineering. Engineering ethics arose as a philosophical discipline in the United States during the 1970s and was initially conceived as “ethics in engineering,” meaning simply ethics for professional engineers (Martin & Schinzinger, 1983). For Li Bocong, however, engineering is more than a profession, and his engineering ethics involves more than strictly professional responsibilities.

In the United States especially the ethics of professional engineers has no doubt made practical contributions to the profession. It has effectively enhanced the ethical consciousness of the professional engineering community, as well as promoted ethics education for engineering students. For Li Bocong, however, the limitations of this approach can be illustrated by considering the question of the impact of engineering on the environment. Engineers do have inescapable responsibilities for environmental pollution. But other relevant actors (such as entrepreneurs or managers) and institutions are also accountable. Indeed, even in the United States there are scholars who have argued that engineering ethics should go beyond individualist ethics to collective, social, or macro ethics (Devon and van de Poel, 2004; Herkert, 2004). Li sees this response as only partially adequate (2008, 2012).

For Li Bocong engineering is best understood not in terms of professional engineers but in terms of the engineering project. An engineering project involves such different moments as task initiation, designing, manufacturing or constructing, implementing, and maintaining. The actors engaged across the course of such an engineering project include not only engineers but also politicians, investors, workers, decision-makers, managers, salespersons, consumers, and more—the collection of which Li defines as an engineering community. According to Li, engineering ethics could and should shift from a limited focus on the professional ethics of engineers to group or collective ethics for the engineering community.

When engineering is taken in Li Bocong’s broad sense as constituted by the heterogeneous community of persons involved in an engineering project, the central topic in engineering ethics ceases to be the behavior of professional engineers and becomes instead focused on decision- or policy-making and the process of integrating such diverse factors as scientific knowledge, technological invention, economic viability, effective management, social acceptability, and a host of institutional, cultural, and environmental constraints. Accordingly, engineering ethics is then the study of the decisions, policies, and values that are morally desirable in engineering projects.

To integrate different interests and successfully realize engineering projects requires coordination. Coordination is the most commonly used method in engineering project activity and decision-making. Deliberate and wise coordination integrates economic, technological, political, environmental, ethical, and other relevant factors. In this sense, engineering ethics is necessarily the ethics of coordination. Li Bocong makes at least five observations relative to an ethics of coordination.

First, there is something distinctly inadequate about the moral universalism characteristic of both deontological and utilitarian engineering ethics in relation to coordination ethics. At least this has been the experience of Chinese engineers faced with the deontological and utilitarian codes of engineering conduct formulated in the West. Engineering involves particularity in both persons and projects. Any coordination ethics concerns a particular subject taking into account the particularities of a specific engineering project. Coordination in dam construction can be quite different from coordination in computer construction, if for no other reason than the projected time scale of use.

Second, engineering activity is not purely ethical, but is the combination of ethical and diversified non-ethical elements that must be coordinated for successful engineering projects. It is not advisable for engineering decision-makers and ethicists to adopt a standpoint or method that overemphasizes or exaggerates any one element, such as economics, technology, politics, social acceptability, ethics, or anything else.

Third, the basic theme of engineering ethics is not some good or right separable from that of the engineering community itself, which often seems so important to much traditional Western ethics. Engineering and coordination in engineering necessarily takes place under restrictions or limits of finite human life, constrained natural resources, and restrictions of physical and social space and time.

Fourth, the term “rationality”, which is again so important to Western ethics, has been challenged by research in economics, sociology, psychology, and political science. Based on the ideal of “perfect rationality”, which conceives of individuals as acting so as to maximize utilities, rationalist ethics aims for universal, irreplaceable, optimal, or invariable decisions. By contrast, the concept of “bounded rationality” counsels the pursuit of satisfactory solutions (Simon, 1957), an approach much more appropriate to coordination ethics.

Finally, fifth, in contrast with the universal propositional and personal tacit knowledge found in Western philosophy, the former appears less and the latter more relevant to an ethics of coordination. The opposite would seem to be the case in ethics as conceived in much traditional ethical theory, at least in Li Bocong’s view.

## Conclusion

Li Bocong has developed one of the most comprehensive philosophies of engineering and technology, and one that exhibits characteristics often quite different than those advanced by Western philosophers. The recent publication of a volume that collects and revises earlier translations of a suite of Li’s studies (Li, 2021) provides an opportunity to broaden appreciation of Li’s contributions to the field. Three specific challenges that we submit are relevant today are Li’s argument for a more expansive understanding of engineering, one that does not limit itself to technical design; the need for a sociology of the engineering community; and a more expansive conception of engineering ethics, one not limited to professional responsibility. With regard to all three elements, we submit that Western philosophers of engineering and technology have much to learn from the long, productive, scholarly career of Li Bocong.
